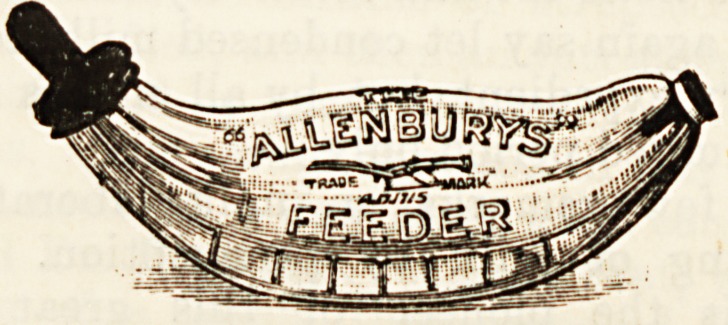# Lectures on the Feeding of Infants

**Published:** 1905-01-07

**Authors:** James Burnet

**Affiliations:** Registrar Royal Hospital for Sick Children; Senior Clinical Tutor, Extramural Wards Royal Infirmary; Physician to The Marshall Street Dispensary, Edinburgh


### Hospital Clinics.

### LECTURES ON THE FEEDING OF INFANTS.

By James Burnet, M.A., M.B., M.R.C.P., Edin. Registrar Royal Hospital for Sick Children; Senior Clinical Tutor, Extramural Wards Royal Infirmary; Physician to The Marshall Street Dispensary, Edinburgh.

### II.—Artificial Feeding.

When the infant is not thriving upon breast milk, or when the mother is unable for one of the reasons referred to in our last lecture to nurse her child, then artificial or hand-feeding must be resorted to. Unfortunately this method of feeding is often most unsatisfactory, even when properly carried out. The physician has to give each individual case special attention, as no two infants will be found to gain weight under the same method of feeding. Each has its own little idiosyncrasies, and these must be studied with extreme care, otherwise hopeless failure will result.

Before considering the question of a substitute for breast milk we shall briefly refer to the subject of feeding-bottles. These are now manufactured in different forms, but we may roughly divide them into two classes—(*a*) those with a long rubber tube, and (*b*) those with a simple teat. The former need not further be considered, as this pattern is quite unsuitable, for the simple reason that the tube cannot possibly be kept clean, and also because the parent or nurse is enabled by using it to neglect the feeding of the infant altogether, who is left to take his bottle as he lies in his cot. The second class of infant feeding-bottles has now quite a large number of different patterns belonging to it, but the Allenbury type here illustrated is, we believe, one of the best obtainable. It has the advantage of possessing a through channel, so that the bottle can be flushed out after use. This is the bottle we personally prefer, and our readers will find that its adoption leads to good results.

The simplest substitute for the breast is cow's milk. This, however, differs very markedly from human milk, as the following table at once shows. The figures represent percentage composition.

| — | Human. | Bovine. | | | |
|---|---|---|---|---|---|
| | Normal Average. | English. | American. | French. | German. |
| Fat ... | 3·30 | 3·70 | 3·75 | 4·00 | 3·69 |
| Sugar ... | 6·80 | 4·48 | 4·42 | 5·00 | 4·88 |
| Proteids ... | 1·50 | 4·06 | 3·76 | 3·40 | 3·55 |
| Ash ... | 0·20 | 0·76 | 0·68 | 0·60 | 0·71 |
| Water ... | 88·20 | 87·00 | 87·39 | 87·00 | 87·17 |

The facts we gather from this table are that (1) the amount of fat in breast milk is practically equal in amount to that contained in cow's milk; (2) the amount of sugar in breast milk is in excess; and (3) the ash or mineral matter is slightly less in amount. The ingredients, however, which shows most marked difference are the proteids. This fact is one of the greatest importance, for on it hinges the essential difficulty in feed-ing infants on cow's milk which contains two-and-a-half times as much proteid as breast milk does. Moreover, the casein of breast milk and that of bovine milk differ very materially. The latter, when taken into the stomach, forms small soft coagula which are readily acted on by the gastric secretion, whereas the casein of bovine milk forms in the infant's stomach large and tough masses which are so difficult of digestion, and hence they are often passed as curds in the stools of the infant.

Another fact requiring attention is that although breast milk is absolutely sterile, cow's milk is crowded with micro-organisms. It is often said that human milk is alkaline, whereas cow's milk is acid, but in reality this statement is not quite accurate, as cow's milk only becomes acid after it has been exposed to the air for some time, but when quite freshly drawn it is either alkaline or neutral in reaction.

In order to reduce the amount of proteid cow's milk must be diluted before use. This dilution, however, reduces the percentage both of the fat and also of the contained sugar. Accordingly, before being given to the infant, the diluted milk must be enriched by the addition of cream and of milk sugar in sufficient amounts. The diluent most commonly employed is water, but in many cases barley-water or even lime-water is to be preferred. Roughly speaking a teaspoonful of cream is sufficient for each feeding, and half a teaspoonful of milk-sugar should be added to each bottle. The dilution requires to be carefully regulated according to the infant's age, and also according to his digestive power. Where the latter is feeble a greater degree of dilution will usually be found advantageous. The following table indicates the degree of dilution we generally observe.

| | Milk. | | Diluting fluid. |
|---|---|---|---|
| First week ... ... ... | 1 | ... | 3 |
| Second week ... ... ... | 1 | ... | $2\frac{1}{2}$ |
| Third week ... ... ... | 1 | ... | $2\frac{1}{4}$ |
| Fourth week ... ... ... | 1 | ... | $2\frac{1}{4}$ |
| Second and third months ... | Rather more of diluting fluid than of milk. | | |
| After the third month ... | Equal parts. | | |

The use of cow's milk in the way we have described is generally followed by successful results if care is taken in the preparation of the milk mixture.

We shall now briefly refer to the various means which have been suggested with a view to rendering cow's milk germ free. These are practically three in number:—

(a) *Sterilisation.*—The milk is brought to the boiling point, and is then kept boiling for 15 to 30 minutes. It is then removed from the steriliser and allowed to cool. Complete sterilisation cannot be obtained at this temperature in less than from one-and-a-half to two hours.

(b) *Pasteurisation.*—The milk is here raised to a temperature of at least 158° F., and then cooled rapidly. It ought to be maintained at the necessary temperature, which is given variously by different writers, for at least half-an-hour. By this means the infectivity of any tubercle bacilli that may be present is diminished and all spore-bearing organisms are devitalised when the milk has been kept at this temperature for five or ten minutes. The method is not absolutely reliable, and is quite ineffectual when the milk has been obtained from a tuberculous source.

(c) *Simple Boiling.*—This is often otherwise termed "scalding." The milk is brought almost to the boiling point, and by this means it would appear that the digestibility of the milk is rendered easier, while at the same time certain of the pathogenic organisms usually present in milk are destroyed.

Personally we prefer the last mentioned method. It produces a looser milk-clot in the infant's stomach, and consequently renders digestion of the proteid material less troublesome. It, however, interferes to some extent with the assimilation of the fat, the globules of which are apt to become surrounded by a proteid envelope which is not readily disintegrated in the infant's stomach. The chief objection to Pasteurisation is that it does not destroy all the pathogenic organisms present in milk, and that milk thus treated will only remain pure for a very short time. Sterilisation of milk is a question which has always aroused keen controversy, some authorities favouring its adoption and others speaking strongly against it. For our own part we do not advise it, as we have found that the use of sterilised milk for any length of time leads undoubtedly to the production of rachitis and of scorbutus as well as of constipation, anæmia, and imperfect development. This is probably owing to interference with fat assimilation, and also to a slight extent because of the destruction of some of the milk-sugar which undergoes a decided change during the process.

Instead of cow's milk that of the ass or of the goat is sometimes employed. Ass's milk contains little fat and a low percentage of proteid, and is specially suited to the needs of infants during the first two or three months. Goat's milk closely resembles cow's milk in composition and possesses nothing of advantage over it. The curd of goat's milk is said to be looser, but we very much doubt this statement.

It frequently happens in practice that the infant, even after a prolonged trial, fails to thrive on diluted cow's milk. The reason for this is usually found to lie in the inability of the infant to digest the proteid of the milk. To get over this difficulty whey may be tried. The best method of preparing whey is to coagulate milk with rennet. Thereafter the curd should be thoroughly separated with the handle of a spoon and the whole squeezed through muslin. The resulting liquor should again be subjected to filtration in order to remove any curd that may have got through the meshes of the muslin. The analysis of such a preparation of whey is somewhat as follows:

| | | |
|---|---|---|
| Fat ... ... ... ... ... | 1·00 | per cent. |
| Sugar ... ... ... ... ... | 4·79 | " |
| Proteid ... ... ... ... ... | 0·86 | " |
| Ash ... ... ... ... ... | 0·15 | " |
| Water ... ... ... ... ... | 93·20 | " |

It will be observed that the object desired is thereby obtained, namely, the production of food material which contains a small amount of proteid. This fluid we have found admirably suited to the requirements of infants. It may be diluted with barley water as required, and cream may be added if thought necessary and desirable.

*Peptonised milk* is sometimes of value. By peptonising the milk the infant is able to take a greater amount of proteid material than it can when the milk is simply diluted. Peptogenic milk powder, which consists largely of lactose, may conveniently be employed for this purpose. It is supplied in bottles, and directions are given on the label so that we need not repeat them here. The only drawback to its use is the expense which makes it unsuitable for use in a working-class practice. Peptonised milk should not be given for any length of time, otherwise the infant's digestion will tend to become weakened and impaired.

We have reserved for final consideration the use of condensed milk as a substitute for breast feeding. We admit that its use is quite allowable for temporary purposes in order to tide the child over its digestive troubles and to serve as a stepping-stone to more nutritious forms of infant food, but we absolutely condemn its constant employment as thereby rachitis, with all its attendant dangers, is sure to be induced, even although the infant appears to be fat and flourishing. Either the Milkmaid brand or Nestlé's may be employed, but in any case it is well to add cream to every bottle in order to avoid the danger of fat-starvation. When condensed milk is diluted, as it should be, with at least from ten to twelve parts of water, it is found to contain far too little fat and relatively too much sugar. We would again say let condensed milk be given as a temporary expedient, but by all means restrict its use for a limited period only.

Just a few remarks on milk laboratories and the ordering of milk by prescription. Rotch of America is the pioneer of this great enterprise which has done so much to revolutionise the whole system of infant feeding. The ingredients of the milk mixture are combined so as to resemble the average percentage composition of breast-milk at different periods during the nursing stage. The infant's age and weight are entered on the prescription and then the various ingredients are given in percentages as required. The milk may be had sterilised or boiled as may be considered best. Milk laboratories are undoubtedly useful, but they necessitate great intelligence on the part of the medical attendant and of the nurse. Moreover, such milk is apt to be somewhat expensive. Its value, however, during summer weather is undeniably great, as by using laboratory milk we insure a purer supply than can otherwise be obtained. If laboratory milk is used care must be taken not to fall into the habit, so readily acquired, of prescribing stock routine mixtures according to the particular age of the child. In every case the requirements of the particular infant as regards proteid and fat digestion must be fully considered and the prescription modified accordingly. Laboratory milk which has been sterilised is, in our opinion, vastly inferior to milk which has simply been brought to the point of boiling and then carefully bottled after cooling.

## Figures and Tables

**Figure f1:**